# Bioactive Compounds from the Zingiberaceae Family with Known Antioxidant Activities for Possible Therapeutic Uses

**DOI:** 10.3390/antiox11071281

**Published:** 2022-06-28

**Authors:** Raphael N. Alolga, Feizuo Wang, Xinyao Zhang, Jia Li, Lam-Son Phan Tran, Xiaojian Yin

**Affiliations:** 1 Clinical Metabolomics Center, School of Traditional Chinese Pharmacy, China Pharmaceutical University, Nanjing 211198, China; alolgara@cpu.edu.cn (R.N.A.); 3220020270@stu.cpu.edu.cn (F.W.); 3220070753@stu.cpu.edu.cn (X.Z.); 2 State Key Laboratory of Natural Medicines, Department of Pharmacognosy, China Pharmaceutical University, Nanjing No. 639 Longmian Road, Nanjing 211198, China; 3 School of Medicine & Holistic Integrative Medicine, Nanjing University of Chinese Medicine, Nanjing 210023, China; 460184@njucm.edu.cn; 4 Institute of Research and Development, Duy Tan University, Da Nang 550000, Vietnam; 5 Institute of Genomics for Crop Abiotic Stress Tolerance, Department of Plant and Soil Science, Texas Tech University, Lubbock, TX 79409, USA

**Keywords:** antioxidants, biological activities, curcuminoids, gingerols

## Abstract

The Zingiberaceae family is a rich source of diverse bioactive phytochemicals. It comprises about 52 genera and 1300 species of aromatic flowering perennial herbs with characteristic creeping horizontal or tuberous rhizomes. Notable members of this family include ginger (*Zingiber officinale* Roscoe), turmeric (*Curcuma longa* L.), Javanese ginger (*Curcuma zanthorrhiza* Roxb.), and Thai ginger (*Alpinia galanga* L.). This review focuses on two main classes of bioactive compounds: the gingerols (and their derivatives) and the curcuminoids. These compounds are known for their antioxidant activity against several maladies. We highlight the centrality of their antioxidant activities with notable biological activities, including anti-inflammatory, antidiabetic, hepatoprotective, neuroprotective, antimicrobial, and anticancer effects. We also outline various strategies that have been applied to enhance these activities and make suggestions for research areas that require attention.

## 1. Introduction

During aerobic metabolism, it is inevitable for cells to generate free radicals, particularly reactive oxygen species (ROS), the levels of which are kept in check by an intricate internal antioxidant defense system [1,2]. In the event that the homeostasis of pro-oxidation and anti-oxidation is broken, oxidative stress will occur [3]. Cellular and subcellular damage caused by oxidative stress has been identified as a cardinal pathological event in the etiologies of various diseases, including, but not limited to, inflammatory diseases [4], cardiovascular diseases [5], cancer [1,6], and the aging process [7]. The cellular effects of ROS engender lipid peroxidation, leading to the production of lipid peroxides, which are known to have deleterious effects on the cell and its composition [8]. The actions of ROS, such as superoxide anions, hydrogen peroxides, and hydroxyl radicals, result in pathophysiological events such as diabetes, ischemia, and inflammatory diseases [9,10]. To counteract the toxic effects of ROS, cellular antioxidant enzymes, such as glutathione peroxidase (GPX), catalase (CAT), and superoxide dismutase (SOD), act as ROS scavengers [6,10].

In recognition of the axiom, “You are what you eat”, the scientific community has devoted considerable effort to finding natural sources of antioxidants in food. The essence of these exogenous antioxidants is complementing the endogenous antioxidant defense system in removing excess free radicals [11,12]. In this regard, notable products, such as green tea as well as various spices and herbs, are recognized as good sources of exogenous antioxidants [13,14,15]. These dietary antioxidants are usually phenolic or thiolic compounds, a major source of which is the Zingiberaceae family (also known as the ginger family) [16].

The Zingiberaceae family consists of about 1300 species of aromatic, flowering perennial herbs with characteristic creeping horizontal or tuberous rhizomes [17]. They are widely distributed in three continents: the Americas, Africa, and Asia. The most notable members of this family include ginger (*Zingiber officinale* Roscoe), turmeric (*Curcuma longa* L.), Javanese ginger (*Curcuma zanthorrhiza* Roxb.), and galangal or Thai ginger (*Alpinia galanga* (L.) Willd.) [18,19]. A wide array of bioactive compounds from this family have been isolated and characterized. The common Zingiberaceae products and representative compounds are shown in Table 1. In this review, we will focus on two main classes of compounds that have been extensively researched, namely, the gingerols (and their derivatives) and the diarylheptanoids. Specifically, we will discuss the major bioactivities credited to their antioxidant effects and some other mechanisms, as well as the strategies adopted to date to improve upon these bioactivities. Although diarylheptanoids are largely present in most members of the Zingiberaceae family, the diarylheptanoids of interest in this write-up are, specifically, the curcuminoids curcumin (CUR), demethoxycurcumin (DMC), and bisdemethoxycurcumin (BMDC). Finally, a few suggestions on topical issues are provided for future research. 

## 2. Methodology

**Figure 1:**
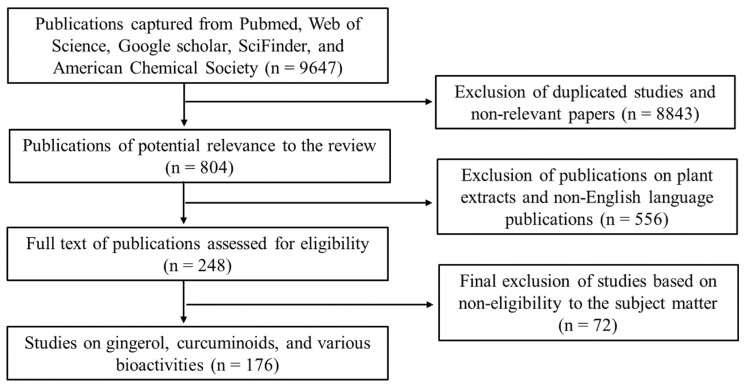
A flow chart of the inclusion and exclusion criteria adopted for screening and selection of publications used in this review.

We sought relevant publications from various scientific platforms, such as Web of Science, Pubmed, SciFinder, American Chemical Society, Elsevier, and Google Scholar using the following keywords: Zingiberaceae; Zingiberaceae and bioactive compounds, Zingiberaceae and bioactivities, Zingiberaceae and antioxidant activities, gingerols and bioactivities, curcuminoids and bioactivities, etc. Publications on extracts of the Zingiberaceae products were excluded, and only publications in the English language were used. Based on the relevance to the subject matters of this review, the publications were scaled down from 533,242 to 176 based on the criteria illustrated in Figure 1. 

## 3. Physicochemical Characteristics

### 3.1. Gingerols and Their Derivatives

The gingerols and their derivatives (basically, polyphenolics in nature) are responsible for the characteristic pungent smell of many members of the Zingiberaceae family, particularly the genus *Zingiber* [24,25]. A notable example is *Z. officinale* Roscoe. Fresh ginger usually contains a high content of 6-gingerol, while other gingerols, such as 4-, 8-, 10-, and 12-gingerol, are present in appreciably good amounts [24,25]. These gingerols are usually converted to their respective shogaols upon drying, long-term storage, or thermal processing. The halogenation of the shogaols produces their corresponding paradols [24,25]. 

**Figure 2:**
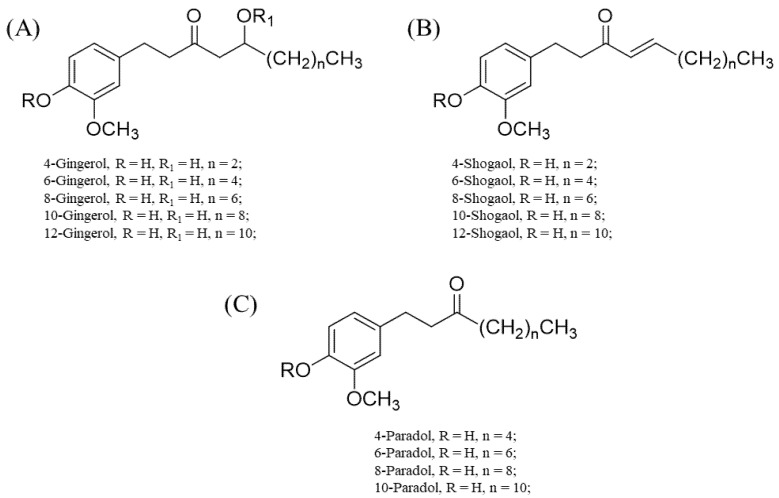
Chemical structures of 4-, 6-, 8-, 10- and 12-gingerols; 4-, 6-, 8-, 10- and 12-shogaols; and 4-, 6-, 8-, and 10-paradols. (**A**) Basic skeletal structure of the gingerols and specific substituents that correlate to each gingerol type. (**B**) Basic skeletal structure of the shogaols with respective substituents for specific shogaol types. (**C**) Basic skeletal structure of the paradols with specific substituents for each paradol type.

The differences in the structural conformation of these compounds (Figure 2) grant them unique and/or enhanced binding affinities to various sites. For instance, structure–activity relationship studies through computational simulations (molecular docking) showed that the linear chain and double bond shared by C4 and C5 in 6-shogaol account for its enhanced activity towards nitric oxide synthase (NOS), cyclooxygenase, and acetylcholinesterase [26,27]. For 6-gingerols, the presence of C=O on C3, the presence of hydroxyl (OH) on C5, and substitution of the linear chain with an aromatic ring that has a hydrogen donor or acceptor at either the meta or para position bonded to it largely account for their notable anti-inflammatory effects (i.e., the inhibition of pro-inflammatory cytokine and cyclooxygenase-2 production) [26,27]. 

### 3.2. Curcuminoids

**Figure 3:**
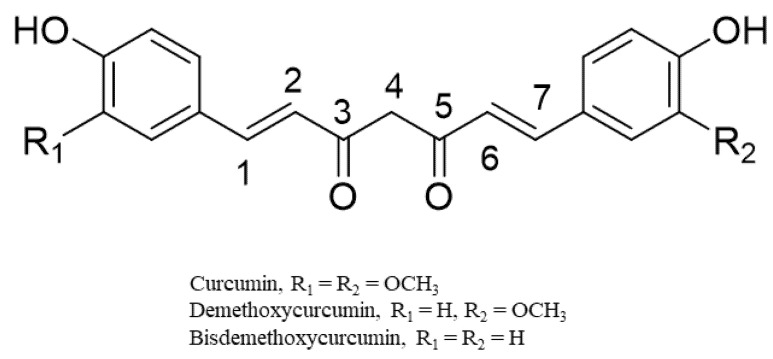
Representative chemical structures of the curcumin, demethoxycurcumin, and bisdemethoxycurcumin.

The curcuminoids (CUR, BDMC, and DMC) belong to diarylheptanoids and are credited with numerous bioactivities [28] (Figure 3). They are phenolic compounds that exist in their diketo–enol conformations. The unique chemical structure of these compounds has bestowed on them diverse pharmacological activities [28]. The main underlying mechanisms of these biological activities are mainly derived from the antioxidant potentials of the compounds [29,30]. Chemical modifications of the structures of the curcuminoids to enhance their biological activities have also been reported, especially for the most abundant component, CUR [31,32]. From an examination of the structure of CUR, it is evident that CUR possesses two phenyl rings with methoxyl and hydroxyl groups that are linked by a keto–enol linker composed of seven carbons. Structure–activity relationship studies on the derivatives of CUR highlight the relevance of a coplanar hydrogen group as well as a diketone moiety for specific bioactivities (e.g., antiandrogenic activity) [29,30]. The introduction of a methyl group to C2 and C6 effectively increased its antiproliferative effect in vitro and in vivo. The hydrogenation, methoxylation, and unsaturation of the diketone moiety of CUR have been linked to increased anti-inflammatory, antioxidant, and anticancer activities of its derivatives [29,30]. 

## 4. Notable Bioactivities

### 4.1. Antioxidant Activities

CUR is a good inhibitor of lipid peroxidation as shown in vitro using human erythrocyte membranes and rat liver microsomes [31,33]. In vivo research involving rat brain, liver and kidney shows consistent results [34]. CUR also inhibits superoxide anion generation in the xanthine–xanthine oxidase system, as well as hydroxyl radical production [30,35,36]. DMC and BDMC are reported to be almost as effective as CUR in their inhibition of iron-induced lipid peroxidation in rat liver microsomes and rat brain homogenate [33,37]. An in vitro comparison of the free-radical-scavenging abilities of the curcuminoids showed that CUR possesses the highest potency, followed by DMC and BDMC [31]. This trend provides evidence in support of the assertion that the methoxy group is crucial for the antioxidant activities of curcuminoids. The curcuminoids possess the ability to protect plasmid pBR322 DNA against the action of a potentially genotoxic ROS, namely singlet oxygen [38]. In this regard, CUR is, again, the most potent inhibitor of DNA damage, relative to DMC and BDMC. This superior property of CUR is partly credited to its unique chemical structure as discussed earlier. In vivo, the antioxidant activities of the curcuminoids are due to their ability to inhibit lipid peroxidation, possibly by ‘mopping up’ oxygen free radicals and augmenting the potential of endogenous antioxidant enzymes. 

The in vitro and in vivo antioxidant properties of gingerols have been investigated by several researchers. In particular, 6-, 8-, and 10-gingerol and 6-shogaol have shown significant dose-dependent free-radical-scavenging activities in vitro. The ROS-scavenging ability of 6-gingerol in B16F10 murine melanoma cells and transforming growth factor beta-1(TGF-β1)-derived nasal polyp-derived fibroblasts was demonstrated by Park et al. [39] and Huang et al. [40]. The 6-gingerol was also demonstrated to dose-dependently inhibit nitric oxide (NO) production and significantly reduce inducible NOS (iNOS) levels in lipopolysaccharide-stimulated macrophages [41]. The antioxidant activities of the gingerols, to a large extent, contribute to the known anti-inflammatory activities of the gingerols (Table 2). In addition to acting as exogenous antioxidants, both curcuminoids and gingerols can activate endogenous antioxidant pathways, such as nuclear factor erythroid 2-related factor 2 (NRF2)- Kelch-like epichlorohydrin -associated protein 1 (KEAP1) pathway [42,43].

### 4.2. Anti-Inflammatory Effects

Since the generation of ROS by activated macrophages is a crucial precondition for the initiation of the cascade of inflammatory events, the abilities of the curcuminoids to scavenge ROS make them good anti-inflammatory agents [44]. In humans, polymorphonuclear leukocytes (PMNLs), which play a pivotal role in the innate immune system, are also critical in the inflammatory process. PMNLs mediate the stage of inflammation when vascular responses are translated into tissue injury. PMNLs produce molecular oxygen derivatives after phagocytosis, which result in a metabolic burst and assist in the functioning of the immune system. When recruited at the inflammatory sites, the PMNLs also release mediators of inflammation, including leukotrienes, ROS, and proteolytic enzymes. The leukotrienes, in particular, are involved in numerous allergic and inflammatory conditions, including allergic rhinitis, psoriasis, and asthma. Any compound with the ability to abort the biosynthesis of leukotrienes, especially 5-lipoxygenase, a key enzyme in the biosynthetic pathway, is worth considering [45,46]. CUR, DMC, and BDMC are reported to exert their anti-inflammatory effects via a plethora of mechanisms, such as the inhibition of NOS induction, scavenging of NO [47], inhibition of 5-lipoxygenase [48], and holistic reduction of oxidative stress [48,49,50], as summarized in Table 2. The gingerols have been variously investigated in vitro and in vivo for their potent anti-inflammatory effects [41,51,52,53]. It is worth mentioning that many of the numerous biological activities they are endowed with, such as antidiabetic and anticancer effects, stem from their ability to mitigate inflammation. 

### 4.3. Antidiabetic Effects

Recent scientific findings on type 1 and type 2 diabetes implicate chronic inflammation in the pancreatic islets as a common pathophysiological event shared by both types [43,111,112]. The chronic activation of the innate immune system in response to inflammation eventually leads to the impairment of insulin secretion and action and the complications that result therefrom [113]. A recent review article outlined the link between inflammation and type 2 diabetes, a metabolic disorder that largely results from obesity [112]. Chronic low-grade inflammation from obesity is the result of sustained hypoxia [113,114]. Hypoxia in adipose tissues is a precondition for the activation of hypoxia-inducible factor-1α (HIF-1α) and the transcription of genes encoding glucose transporter, vascular endothelial growth factor, and erythropoietin [112,114,115]. The inhibition of HIF-1α represses obesity and ameliorates insulin resistance (i.e., increases insulin sensitivity). The physiological states of hyperlipidemia and hyperglycemia induce the production of ROS, which, in turn, has a cascading effect on other signaling pathways, such as nuclear factor kappa B (NF-κB) activation, leading to the release of pro-inflammatory cytokines, chemokines, and leukocyte adhesion molecules [112]. Under normal glycemic conditions, the binding of insulin to its receptors on the adipocytes activates two main signaling pathways: the phosphatidylinositol 3-kinase-protein kinase B (PI3K/AKT) and the Ras-mitogen-activated protein kinase (MAPK) pathways. The normal functions of these pathways are hampered under hyperglycemic and/or hyperlipidemic conditions due to the release of adipokines and free fatty acids [112,116].

The gingerols have been reported to be good candidates for the treatment of diabetes due to targeting cardinal stages in the etiology of the disease and halting disease progression. A few of the mechanisms to which their antidiabetic potential is ascribed include the followings: the regulation of oxidative stress and inflammation [54], promotion of glucose utilization in adipocytes and myotubes [55], inhibition of hyperglycemia [56], alleviation of hyperglycemia via the Nrf2-mediated pathway [57], potentiation of glucagon-like protein-1-mediated glucose-stimulated insulin secretion in β-cells [58], promotion of membrane presentation of glucose transporter 4 (GLUT 4) in skeletal muscle [64], inhibition of ROS/NF-κkB/cyclooxygenase-2 (COX-2) activation [59], activation of the PI3K–AKT– endothelial NOS pathway [60], dual regulation of glucose metabolism via the adenosine monophosphate-activated protein kinase (AMPK)α2-mediated AKT substrate of 160 kDa-Ras related protein Rab-5A (AS160–RAB5) pathway and AMPK-mediated insulin-sensitizing effects [61], and inhibition of NACHT, LRR and PYD domains-containing protein 3 (NLRP3) inflammasome activation and interleukin-1β secretion [62] (summarized in Table 2).

In a similar vein, the curcuminoids have been demonstrated to possess the antidiabetic potential and ameliorate complications of the disease via a myriad of mechanisms, including, but not limited to, reducing insulin resistance and blood lipids [63,64,65], the modulation of the innate immune system [66], the alleviation of oxidative stress and inflammation [67], the promotion of autophagy and the alleviation of apoptosis (in the case of cardiomyopathy) [68,69], the inhibition of podocyte mesenchymal transdifferentiation and induction of autophagy (for diabetic nephropathy) [70], the modulation of adipokines [71], and the inhibition of the protein kinase C beta (PKCβ) type axis and activation of forkhead box protein O3 (FOXO-3a) (for reversal of diabetic nephropathy) [72]. CUR remains the most researched of the three curcuminoids as a potential antidiabetic agent due to its superior therapeutic qualities.

### 4.4. Hepatoprotective Effects

The antioxidant and anti-inflammatory properties of the curcuminoids have been demonstrated to offer protection against liver damage in different in vitro and in vivo models, such as non-alcoholic fatty liver disease (NAFLD), and carbon tetrachloride (CCl_4_)- and alcohol-induced hepatic damage [117,118,119,120]. The hepatoprotective effects of these compounds are achieved by activating various anti-inflammatory pathways and processes that underlie liver damage, including the alteration of liver lipid and bile acid excretion [117,118]. These effects are at least partly credited to their antioxidant capacities with respect to augmenting the internal antioxidant defense system, preventing oxidative stress and the cascade of inflammatory events therefrom [119]. In in vivo models, such as CCl_4_-induced liver damage, the injection of CCl_4_ produces the free radicals trichloromethyl (^•^CCl_3_) and trichloromethyl peroxy (^•^OOCCl_3_) after hepatic metabolism [120]. These free radicals initiate the release of ROS in the Kupffer cells, which sets off a cascade of events that eventually result in oxidative stress and the release of various cytokines [116,120]. The NAFLD model is usually achieved by feeding laboratory animals a high-fat diet over a defined period, leading to the accumulation of free fatty acids, endoplasmic reticulum (ER) stress, and lipotoxicity [67,118]. These conditions provide a microenvironment conducive to the initiation of the inflammatory process [115,119].

The curcuminoids, therefore, act by blocking or retarding various processes that lead to oxidative stress and inflammation, for instance, by lowering lipid peroxidation and enhancing the capacity of the internal antioxidant defense system [73,74,75,76]. Similarly, the gingerols interfere with different inflammatory pathways, ameliorating various forms of liver damage. For instance, the hepatoprotective effects of 6-gingerol and 6-shogaol are partly ascribed to their abilities to activate the Nrf2 pathway [77], modulate oxidative stress [78], regulate lipogenesis, fatty acid metabolism, oxidative stress, and mitochondrial dysfunction [79], and generally regulate key genes related to inflammation and lipid metabolism [80]. A summary of these mechanisms is provided in Table 2.

### 4.5. Neuroprotective Effects

Neuroinflammation underlies several neurodegenerative diseases, such as Parkinson’s disease, Alzheimer’s disease, and multiple sclerosis [121,122]. The response to neuroinflammation is usually mediated by the macrophages of the central nervous system, namely the microglial cells that are activated in the event of tissue damage or invasion by foreign bodies (e.g., pathogens). The activation of the microglial cells initiates the secretion of different proinflammatory agents such as NO, cytokines (tumor necrosis factor-α, and interleukin-1β), arachidonic acid, and ROS. The action of some pro-inflammatory factors on the neurons initiates apoptosis, while others, such as tumor necrosis factor and interleukin-1β intensify the inflammatory process and disease state by affecting astrocytes and microglial cells [123,124]. A proposed and promising approach for treating neurodegenerative diseases is preventing the initiation of the inflammatory process or completely halting its progression.

Gingerols—specifically, 6-gingerol, 10-gingerol, and 6-shogaol—have been found to elicit neuroprotective effects via diverse mechanisms. These include the suppression of astrocyte overactivation [81], regulation of the mircroRNA-103/B cell lymphoma-2/adenovirus E1B 19 kDa protein-interacting protein 3 (miR-103/BNIP3) pathway [82], modulation of neuroinflammation [83], regulation of the miR-210/brain-derived neurotrophic factor axis [84], regulation of the AKT-Serine/threonine-protein kinase mammalian target of rapamycin (mTOR) signal transducer and activator of transcription 3 (STAT3) pathway [85], inhibition of NLRP3 inflammasome activation and apoptosis [86], and fortification of the cellular antioxidant defense system [87]. The curcuminoids have been found to possess various neuroprotective potentials, largely by attenuating oxidative stress and the actions of inflammatory cytokines [88,89]. In the brain, they prevent β-amyloid accumulation and/or aggregation, and oligomer-dependent Aβ toxicity [90,91]. The curcuminoids also mitigate iNOS, COX-2, TGF-β1/2, matrix metalloproteinase-9 (MMP-9), and brain-derived neurotrophic factor (BDNF) expression [92], attenuate α-synuclein aggregation [93], and generally, lessen the severity of the symptoms of neurodegenerative diseases [94] (Table 2).

### 4.6. Anticancer Activities

The interplay between inflammation and cancer has been greatly clarified over the past century [125,126,127,128,129]. Inflammation remains a central characteristic of tumor progression. The tumor microenvironment, which is mainly an orchestration of various inflammatory cells, such as monocytes, dendritic cells, natural killer cells, macrophages, neutrophils, and T-lymphocytes, is a critical player in the proliferation, survival, and migration of neoplastic cells [125,129]. Chronic inflammation owing to incessant microbial infections, infections from helminths, or even exposure to non-infectious materials, such as silica, asbestos, or smoke, can eventually lead to carcinogenesis [129]. Chronic low-grade inflammation induced by obesity promotes a variety of cancer as well [126,127]. It is also known that under the influence of the inflammatory microenvironment, tumor cells tend to co-opt innate immune system signaling molecules such as the chemokines and selectins, as well as their receptors, for the purposes of invasion, migration, and metastasis [129]. Moreover, the production of tumor necrosis factor related apoptosis-induced ligand (TRAIL) cytokines and their binding with death receptors trigger a cascade of apoptotic events [129].

On the basis of the aforementioned information, the role of anti-inflammatory agents in the treatment of cancers cannot be gainsaid. The curcuminoids and gingerols (and their derivatives) with proven antioxidant, anti-inflammatory, antimicrobial, and antiviral activities have been investigated as possible candidates for the management and/or treatment of various cancer types. Among the curcuminoids, CUR has been proven to exhibit, by far, the best activity against cancers, such as breast, lung, hematological, gastric, colon, pancreatic, and hepatic cancers [95]. Various review articles on CUR and cancer abound. A recent review by Giordano and Tommonaro delved into the intricacies of the role of CUR in the various cancer types is worth reading, since much of the information presented therein falls outside the remit of this review [95]. Another review of relevance to the subject matter of cancer and the curcuminoids worth considering is that of Tomeh et al. [96]. Moreover, a randomized controlled trial by Panahi et al. found that, after 8 weeks of curcuminoid supplementation (500 mg/day), the erythrocyte sedimentation rate, serum levels of C-reactive protein, and general quality of life of stage 3 colorectal cancer patients were improved [97].

Gingerols, specifically, 6-, 8- and 10-gingerol and 6-shogaol, have shown varying levels of activity against colorectal, breast, gastric, cervical, ovarian, prostate, and lung cancers in vitro and in vivo. These anticancer activities are mainly based on their inhibition of the different stages of cancer [98]. The gingerols modulate various signaling pathways related to cancer, such as NF-κB, signal transducer and activator of transcription 3 (STAT3), MAPK, activator protein-1 (AP-1), β-catenin, proinflammatory mediators (tumor necrosis factor-α and COX-2), and growth factor receptors (epidermal growth factor receptor and vascular endothelial growth factor receptor) [98]. There is, however, a paucity of information on clinical trials of gingerols. One of the few clinical studies on the gingerols, specifically, 6-gingerol, was conducted by Konmun et al. [99]. This phase 2, randomized, double-blind, placebo-controlled study sought to examine the anti-emetic activity of 6-gingerol (10 mg) taken twice daily for 12 weeks in cancer patients on emetogenic chemotherapy. They found that the administration of 6-gingerol significantly improved chemotherapy-induced nausea and vomiting, appetite, and the general quality of life of the cancer patients undergoing chemotherapy (Table 2) [99]. When treated with heat stress, gingerols are converted into shogaols with enhanced anticancer and anti-inflammatory effects. Moist heat treatment is an ideal method to obtain a high quantity of bioactive components of shogaols [130,131]. Hence, consuming ginger as tea is a good choice.

### 4.7. Antimicrobial Properties

Despite extensive research, the search for new and efficacious antimicrobial drugs with few side effects of natural origin is still ongoing. This is primarily necessitated by the emergence of and surge in the incidence of drug-resistant antimicrobial strains. The purified curcuminoids were shown to demonstrate good antimicrobial activities against eight bacterial strains: *Streptococcus agalactiae*, *Staphylococcus intermedius*, *S. epidermidis*, *S. aureus*, *Aeromonas hydrophila*, *Bacillus subtilis*, *B. cereus*, and *Edwardsiella tarda* [100]. The synergistic effects of CUR with cefixime, cefotaxime, vancomycin, and tetracycline were demonstrated in vivo against clinical isolates of *S. aureus* [100]. Similar findings were reported for CUR in combination with ampicillin, oxacillin, and norfloxacin against methicillin-resistant *S. aureus* [100]. Metal complexes strongly bound to various antimicrobial agents have also shown a synergistic influence by increasing the binding affinities of these agents to the bacterial cell wall. In this regard, curcuminoids, particularly CUR, have been complexed with various metals to achieve the desired synergistic effects. These formulations include curcumin–tannic acid-metal complexes (CUR-TA-Fe II and CUR-TA-Fe III) [101], curcumin–zinc oxide nanoparticles [102], and gallium–curcumin nanoparticles [103]. CUR has been reported to possess varying degrees of activity against the following viruses: high-risk human papillomaviruses, coxsackievirus, hepatitis C, herpes simplex virus type 1, influenza viruses (PR8, H1N1, and H6N1), and transmissible gastroenteritis virus [100]. The antifungal potential of the curcuminoids (particularly CUR) was demonstrated against *Paracoccidioides brasiliensis*, various clinical isolates of *Candida albicans* [104], and dermatophytes [105].

Compared with the curcuminoids, relatively few investigations have been conducted on the antimicrobial activities of the gingerols and their derivatives. Among the reported gingerols, the most potent compound was 10-gingerol. The addition of 10-gingerol synergistically enhanced the antimicrobial effects of different aminoglycosides, such as polymyxin, bacitracin, and arbekacin, against vancomycin-resistant enterococci [106]. Recently, 6-gingerol has been reported to be an excellent addition to isoniazid for the treatment of tuberculosis due to its potent antimycobacterial and immunomodulatory effects against *Mycobacterium tuberculosis* [107]. A combination of 6-gingerol and tobramycin was proved very effective against *P. aeruginosa*, and therefore, holds prospects for clinical applications for the treatment of *P. aeruginosa* infections [108]. In addition, 6-gingerol possesses antiviral effects, specifically against the Chikungunya virus, by inhibiting its replication [109]. The antifungal properties of 6-gingerol and its derivative, 6-shogaol, against fluconazole-resistant *C. albicans* were demonstrated and were found to be caused by their antibiofilm and antivirulence effects [110] (Table 2).

### 4.8. Safety of Compounds from Zingiberaceae

A total of 12 species from Zingiberaceae were included in Compendium of Botanicals and were reported to contain naturally produced substances of possible concern for human health when used in food and food supplements by European Food Safety Authority (EFSA) [132]. There are considerable studies on ginger, which contains a large amount of gingerol. There is no report about oral toxicity and genotoxicity of Zingiberis Rhizoma Recens, the fresh rhizoma from Z. officinale Roscoe [21]. CUR is evaluated as Generally Recognized as Safe (GRAS) by the U.S. Food and Drug Administration (FDA). CUR shows no ability to induce gene mutations or structural chromosomal aberrations [133]. In a 14-day repeated-dose oral toxicity study, the no-observed-adverse-effect level (NOAEL) of CUR is as high as 2000 mg/kg, and in a 90-day repeated-dose oral toxicity study, the NOAEL of CUR is 1000 mg/kg [133]. Both short-term and long-term toxicology research confirm that CUR is safe in a moderate dose [133].

## 5. Enhancement of Bioactivities

As previously indicated, the most active compound among the curcuminoids is CUR. Based on the Biopharmaceutics Classification System, CUR is classified as a Class IV drug; it is characterized by poor water solubility and nominal gastrointestinal epithelial permeability [134]. CUR is also a P-glycoprotein substrate and is thus expelled from the intestinal membrane via the ATP-dependent drug efflux pump [135]. To address the bioavailability challenges for CUR, various strategies have been proposed. Many of these strategies have aimed to improve its solubility via the use of solid self-emulsifying drug-delivery systems, solid dispersions, and cyclodextrin inclusion complexes [136]. Other approaches include the synthesis of prodrugs, solid-state crystal structural manipulation, and micronization to increase the surface area of the drug for dissolution. As it is a P-glycoprotein substrate, a feasible approach to increase the bioavailability of CUR is to inhibit the activity of P-glycoprotein using inhibitors, such as piperine and quercetin [123,136]. Nanoformulations have served as practicable and attractive alternatives for drugs that have bioavailability challenges. The merits of nanoformulations include: (1) the ability to achieve small particle sizes and increase surface areas for nanoparticles for enhancing bioavailability; (2) the ease of transporting a drug load through the gastrointestinal mucosal barrier; (3) the option of obtaining controlled and sustained release formulations; and (4) an avenue for drug localization and cell-specific uptake. Various nanoformulation strategies have been developed for the curcuminoids (especially CUR) in order to circumvent their bioavailability challenges and fully exploit their numerous beneficial pharmacological properties. These strategies are broadly classified as follows: nanosuspensions, lipid-based nanoformulations (liposomes and solid lipid nanoparticles), microemulsions and self-microemulsifying drug-delivery systems, nanoemulsions, and polymeric nanoparticles, among others. These categories of nanoformulations for the curcuminoids have their respective strengths and weaknesses, as detailed by Ipar et al. [135] and Liu et al. [136]. The solubility of CUR increases by 12 folds after being heated. Moreover, CUR is hydrophobic. Therefore, when consumed as a food, turmeric utilized in hot dishes employing oil, such as curry, will have better bioactive effects [137].

Compared with the curcuminoids, the gingerols have not been as extensively formulated into nanoparticles. That notwithstanding, the gingerols, to a large extent, encounter similar bioavailability challenges and would therefore benefit from the merits of nanoformulations. As of now, the literature on the nanoformulations of the gingerols is scarce and limited to 6- and 10-gingerols. Of these, 6-gingerol has been formulated into PEGylated nano-niosomes [138] and as phytosomes complexed with chitosan [139] for enhanced antiproliferative effects against breast cancer and the treatment of respiratory infections, respectively. Through the use of magnetic hydroxyapatite-based alginate polymers, 6-gingerol was formulated as a pH-sensitive drug for controlled and targeted delivery to breast and liver cancer cells. Through the use of pH-sensitive sodium alginate and hydroxyapatite-coated iron oxide nanocomposites, the poorly water-soluble molecules 6-gingerol and curcumin were efficiently loaded for targeted and controlled release [140]. Finally, a 10-gingerol-loaded nanoemulsion was formulated for enhancing activity against triple-negative breast cancer cells [141].

## 6. Conclusions and Future Perspectives

This review on the gingerols and curcuminoids, the two classes of bioactive compounds from the Zingiberaceae family with invaluable therapeutic potential, provides a glimpse into the potential vast array of bioactive compounds in this family. These compounds have also gained much attention from the scientific community. There is, therefore, a need for more attention to be paid to the less explored class of compounds, particularly the derivatives of these compounds, so as to fully exploit their medicinal benefits. This review differs from the previous reviews that focused mainly on either single compounds or just one category of the bioactive compounds.

The dearth of evidence from clinical trials of these compounds is worth paying attention to. The most active compounds could form part of a treatment regimen for chronic diseases, such as cancer or even microbial infections, and be evaluated for their possible additive or synergistic effects. They could therefore be administered to supplement the established treatment protocols, at least for possible palliative purposes. Information on such combinations can be sought from clinical trials. Importantly, vital data on the safety of these compounds could also be obtained from such studies.

Per the scientific report of the European Food Safety Authority, certain botanicals contain substances that could be of possible concern for human health, especially when used in food and food supplements [132]. The chemicals of concern in turmeric, as stated in their compendium, are monoterpene etheroxide (1,8-cineole) and bicyclic monoterpene (camphor) that are found in the essential oil [132]. Although no specific toxic or adverse effects have been linked to these compounds, there is a need for the toxicological (safety) assessments of these compounds. For ginger, no chemical of concern has been identified. However, on the basis of the findings of an in vivo study [142], attention should be paid to the amount of fresh rhizomes consumed or infusions prepared from ginger. These safety concerns for the extracts of turmeric and ginger could be extended to the individual bioactive compounds highlighted in this review. Further studies on the effects of gingerols and curcuminoids in humans are required so as to determine the maximum daily permissible quantities for human consumption.

The outbreak of the COVID-19 pandemic called the attention of scientists to the concept of drug repurposing. Considering the urgency of the situation and the importance of not reinventing the wheel, many researchers have devoted considerable attention to bioactive compounds from plant sources as potential candidates. In computer simulation studies (i.e., molecular-docking studies), CUR, 6-gingerol, 6-shogaol, and 6-paradol were found to possess high to moderate binding affinities to various enzymes and receptors. CUR was found to bind potently to angiotensin-converting enzyme 2 (ACE2), mutated spikes, and mutated spike–ACE2 complexes, possibly restricting viral entry [143]. CUR, 6-gingerol, 6-shogaol, and 6-paradol exhibited moderate binding affinity to and demonstrated moderate inhibition of the M-pro enzyme of the SARS-CoV-2 virus [144]. CUR and 6-gingerol were also found to putatively inhibit cathepsin K, SARS-CoV-2’s main protease, and SARS-CoV 3-C-like protease [145]. Finally, CUR demonstrated a strong binding affinity to host-specific receptors, furin, and ACE2, while 6-gingerol showed strong interactions with spike proteins and the target protein RNA-dependent RNA polymerase (RdRp) [146]. The outcome of all these investigations indicates that whether used singly or in combination, these compounds could be beneficial for combating the COVID-19 virus. These findings, however, require extensive scientific backing, especially through clinical trials. The few clinical trials conducted to date suggest CUR as a good addition to the treatment regimen to aid in the control of inflammatory responses [147] and improve the recovery times of patients with mild to moderate COVID-19 [148].

## Figures and Tables

**Table 1:** Summary of products and compounds from representative plant species from Zingiberaceae.

Representative Plant	Product	Compounds	Reference
*Curcuma longa* L.	Turmeric (rhizome)	CUR, DMC, and BMDC	[20]
*Zingiber officinale* Roscoe	Ginger (rhizome)	6-gingerol and 4-shogaol	[21]
*Elettaria cardamomum* (L.) Maton.	Cardamom (fruit)	1,8-cineole and catechin	[22]
*Alpinia galanga* (L.) Willd.	Galangal (rhizome)	3,5,7-trihydroxyflavone (galangin)	[23]

**Table 2:** Summary of notable bioactivities of the gingerols and the curcuminoids. iNO, inducible nitric oxide synthase; ROS, reactive oxygen species; NF-κB, nuclear factor kappa B; NLRP3, NACHT, LRR and PYD domains-containing protein 3; PPARγ, peroxisome proliferator-activated receptor γ.

Bioactivity	Compounds	Mechanism of Actions	References
Antioxidant	Curcuminoids	Inhibition of lipid peroxidation; ROS scavenging; antioxidant pathway activation	[31,32,33,34,35,36,37,38,42]
	Gingerols	ROS scavenging; inhibition of NO production; significant reductions in iNOS levels; antioxidant pathway activation	[39,40,41,43]
Anti-inflammatory	Curcuminoids	Inhibition of NO synthetase induction; inhibition of 5-lipoxygenase; NO scavenging	[44,45,46,47,48,49,50]
	Gingerols	Regulation of oxidative stress; inhibition of the PPARγ/NF-κB signaling pathway; inhibition of T-lymphocyte proliferation and cytokine synthesis	[41,51,52,53]
Antidiabetic	Gingerols	Regulation of oxidative stress and inhibition of inflammation; promotion of glucose utilization; reduction in hyperglycemia; regulation of glucose metabolism and insulin sensitivity	[54,55,56,57,58,59,60,61,62]
	Curcuminoids	Reduction in insulin resistance and blood lipid levels; alleviation of oxidative stress and inflammation; modulation of innate immune system; modulation of adipokines	[63,64,65,66,67,68,69,70,71,72]
Hepatoprotection	Curcuminoids	Inhibition of inflammation; lowering lipid peroxidation; enhancing the internal antioxidant defense system	[73,74,75,76]
	Gingerols	Inhibition of inflammation; modulation of oxidative stress; regulation of lipid metabolism	[43,77,78,79,80]
Neuroprotection	Gingerols	Modulation of neuroinflammation; inhibition of NLRP3 inflammasome activation and apoptosis; fortification of the cellular antioxidant defense system	[81,82,83,84,85,86,87]
	Curcuminoids	Attenuation of oxidative stress and the actions of inflammatory cytokines; prevention of β-amyloid accumulation and/or aggregation and oligomer-dependent Aβ toxicity; attenuation of α-synuclein aggregation	[88,89,90,91,92,93,94]
Anticancer	Curcuminoids Gingerols	Modulation of various signaling pathways related to inflammation and cancer	[95,96,97] [98,99,100,101]
Antimicrobial	Curcuminoids	Antibacterial activity; antiviral activity; antifungal activity; enhance the inhibitory effect of existing antimicrobial agents through synergism	[100,101,102,103,104,105]
	Gingerols	Antibacterial activity; antiviral activity; antifungal activity; synergistic antimicrobial activity	[106,107,108,109,110]
